# Optimization of measurements with an ultrasound attenuation coefficient algorithm for quantifying liver fat

**DOI:** 10.1007/s00330-025-11906-5

**Published:** 2025-09-05

**Authors:** Giovanna Ferraioli, Davide Roccarina, Takashi Kumada, Sadanobu Ogawa, Yuichi Yoshida, Masashi Hirooka, Richard G. Barr

**Affiliations:** 1 Department of Clinical, Surgical, Diagnostic and Pediatric Sciences, University of Pavia, Campus della Salute, Pavia, Italy; 2 SOD Medicina Interna ed Epatologia, Azienda Ospedaliero-Universitaria Careggi, Firenze, Italy; 3 Sheila Sherlock Liver Unit and UCL Institute for Liver and Digestive Health, Royal Free Hospital, London, UK; 4 Department of Nursing, Faculty of Nursing, Gifu Kyoritsu University, Ogaki, Japan; 5 Department of Imaging Diagnosis, Ogaki Municipal Hospital, Ogaki, Japan; 6 Department of Gastroenterology and Hepatology, Suita Municipal Hospital, Suita, Japan; 7 Department of Gastroenterology and Metabology, Ehime University Graduate School of Medicine, Toon, Japan; 8 Department of Radiology, Northeastern Ohio Medical University, Rootstown, OH USA; 9 Southwoods Imaging, Youngstown, OH USA

**Keywords:** Attenuation coefficient, MRI-PDFF, Liver steatosis, MASLD, Performance studies

## Abstract

**Objectives:**

Methods for measuring the ultrasound attenuation coefficient (AC) vary across different systems. Some have fixed regions of interest (ROI) while others have movable ROIs. Aims were to evaluate whether, using a system with a fixed ROI, correlation between AC and MRI proton density fat fraction (MRI-PDFF), and performance could be improved by (i) reducing fixed ROI length to 30 mm, changing starting point from the transducer, and (ii) using a movable ROI at different depths.

**Materials and methods:**

In this retrospective multicenter study, AC measurements were performed with the Arietta 850 system, and raw data were automatically stored. AC values were obtained using a standard commercial algorithm (ROI-setting1, 35–75 mm from transducer). Raw data were successively reprocessed externally using a fixed 45–75 mm ROI (ROI-setting2) and a movable 30 mm ROI positioned with the top at 20 mm (ROI-setting3) and 25 mm below the liver capsule (ROI-setting4). Spearman rho and area under the receiver operating characteristics curve (AUROC) were used to assess correlation with MRI-PDFF and performance, respectively, and the Delong test was used to compare AUROCs.

**Results:**

Seven hundred fifty participants (median age: 65 [52, 73] years; 384 males) were included. Correlation of ROI-setting1 with MRI-PDFF was 0.75 (0.72, 0.78), reaching 0.80 (0.77, 0.82) with ROI-setting4. Overall, ROI-setting4 showed significantly the best performance across steatosis grades. AUROCs for *S* > 0 were 0.90 (0.87, 0.92) for ROI-setting1 and 0.92 (0.90, 0.94) with ROI-setting4 (*p* < 0.001). This latter performed significantly better than all other settings in participants with obesity and skin-to-liver distance > 25 mm (*p* < 0.05).

**Conclusions:**

A movable ROI improves both AC correlation with MRI-PDFF and performance. The highest improvement was with the ROI top 25 mm below the liver capsule.

**Key Points:**

***Question*** *It remains uncertain whether utilizing an ROI at a fixed position from the transducer is optimal for ultrasound AC measurements*.

***Findings*** *The highest AC performance and correlation with MRI-PDFF were observed with a movable ROI positioned with the top at 25 mm under the liver capsule*.

***Clinical relevance*** *A movable ROI with consistent sub-capsular placement improves both correlation between AC and MRI-PDFF, as well as its performance, whereas in individuals with a skin-to-liver capsule distance > 25 mm, a fixed ROI position significantly decreases AC performance*.

**Graphical Abstract:**

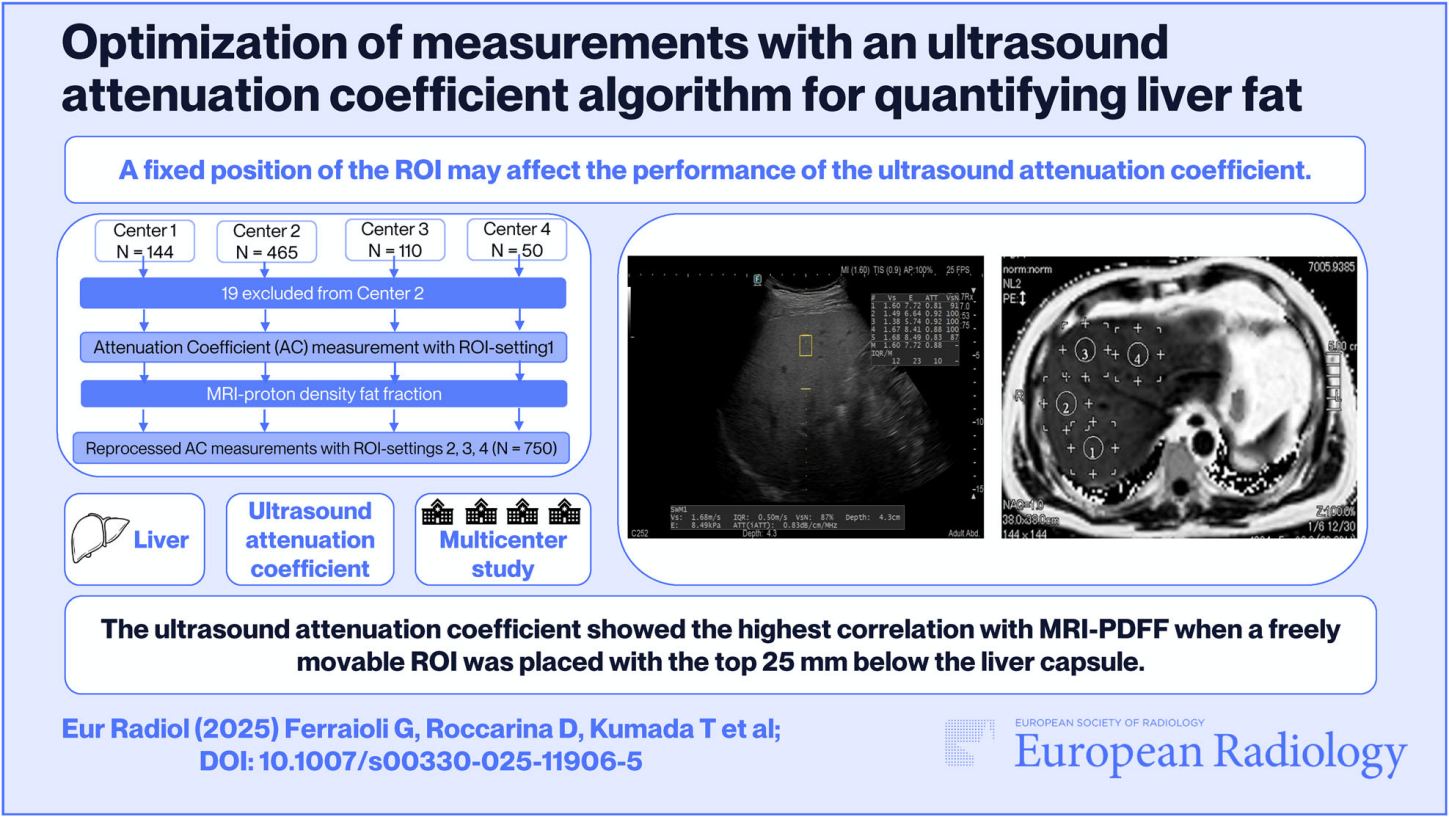

## Introduction

The global prevalence of steatotic liver disease is significant, underscoring the need for a non-invasive and cost-effective method for accurately identifying individuals with liver steatosis. In addition, the FDA approval of the first drug to treat patients with fibrotic metabolic dysfunction-associated steatohepatitis (MASH) [1] will increase the need for reliably assessing changes over time of liver fat content in patients under treatment. Indeed, it has been reported that an improvement in liver steatosis, as indicated by a change in magnetic resonance imaging proton density fat fraction (MRI-PDFF) of ≥ 30%, was associated with histological improvements in MASH [2]. Furthermore, an association has been demonstrated between the amount of fat in the liver and an increased risk of cardiovascular events [3–5]. However, the costs and limited availability of MRI-PDFF must be considered.

Algorithms for the quantification of liver fat content with ultrasound (US) have been developed and are currently commercially available. These algorithms are based on the analysis of the raw data of US signals backscattered from the tissue, and they calculate the attenuation coefficient (AC), the backscatter coefficient, or a combination of both [6].

A depth dependence in the AC estimation has been reported [7]; therefore, it is imperative to standardize the measurement to obtain robust estimates. It is currently unclear whether a fixed position of the ROI can overcome this problem. Indeed, the best method for AC measurement is still an outstanding question, and different manufacturers have adopted different approaches to AC measurement. Some algorithms use fixed regions of interest (ROI) while others have freely movable ROIs. However, the fixed ROI distance may potentially constitute a limitation for individuals with thick subcutaneous tissue, such as those who are overweight or obese. On the other hand, the World Federation for Ultrasound in Medicine and Biology (WFUMB) guidance recommends performing AC measurements with a 30 mm ROI positioned 20 mm beneath the liver capsule [6].

The objectives of this multicenter study were to determine the potential impact on the correlation between AC obtained with integrated attenuation imaging (iATT) and MRI-PDFF and on the diagnostic performance of AC in all participants and in those with obesity or thick subcutaneous tissue (skin-to-liver capsule distance > 25 mm) by (i) reducing the length of the fixed ROI to 30 mm, starting at 45 mm from the transducer, and (ii) enabling the operator to adjust the ROI position freely, allowing measurements at varying depths.

## Materials and methods

This multicenter study was a retrospective evaluation performed by reprocessing prospectively acquired data. It was approved by the Ethics Committee at each participating center, and written informed consent was obtained from all participants. The study was also Health Insurance Portability and Accountability Act (HIPAA) compliant for center4. The study included four cohorts, three in Japan and one in the United States, and enrolled consecutive individuals who agreed to participate. The centers were: Ehime University Hospital, Japan (center1); Ogaki Municipal Hospital, Japan (center2); Suita Municipal Hospital, Japan (center3); and Southwoods Imaging, USA (center4). The study period was from December 2021 to March 2024 at center1; from May 2021 to March 2023 at center2; from February 2022 to March 2023 at center3; and from April to May 2024 at center4.

**Fig. 1:**
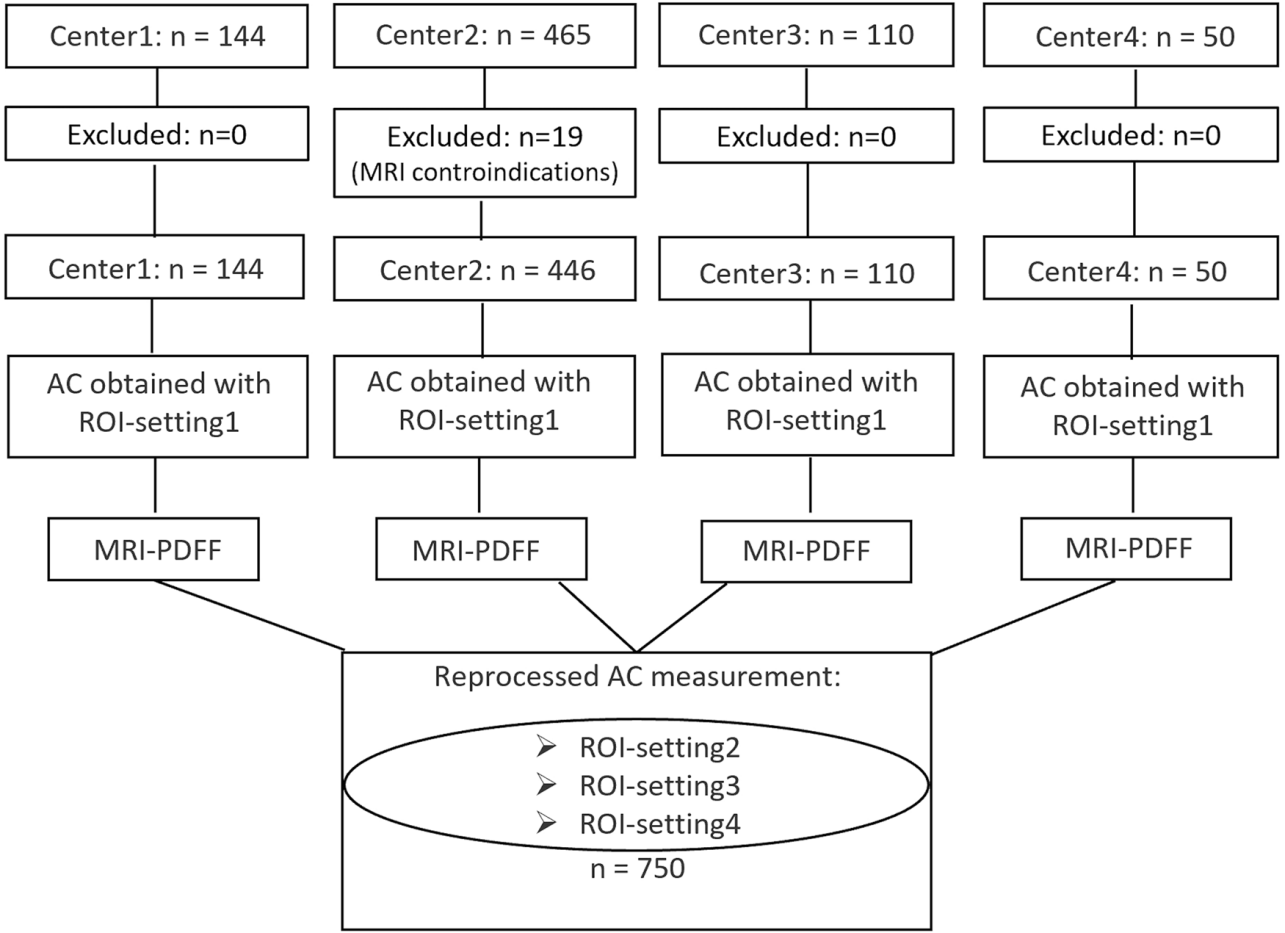
Flow chart of the study. ROI-setting1: fixed size (40 mm) and depth (35–75 mm from the transducer) of the ROI. ROI-setting2: fixed size (30 mm) and depth (45–75 mm from the transducer) of the ROI. ROI-setting3: user-adjustable, 30 mm ROI; ROI upper edge positioned 20 mm below the liver capsule. ROI-setting4: user-adjustable, 30 mm ROI; ROI upper edge positioned 25 mm below the liver capsule. AC, attenuation coefficient; PDFF, proton density fat fraction; ROI, region of interest

Individuals with mixed etiologies of liver disease or at risk of liver steatosis were enrolled. Inclusion criteria included one of the following: (1) known history of steatotic liver disease or chronic liver disease; (2) type 2 diabetes; (3) obesity (body mass index [BMI] ≥ 28 kg/m^2^ in the Japanese cohorts and ≥ 30 kg/m^2^ in the North American cohort). Exclusion criteria were: (1) age < 18 years; (2) segmental heterogeneous steatosis; (3) history of prior liver surgery; (4) claustrophobia; (5) magnetic substances or tattoos in the body; (6) presence of a pacemaker or any other contraindication to MRI; (7) pregnancy; and (8) inability to give informed consent. The flow chart of the study is shown in Fig. 1.

The timeframe between the US and MRI-PDFF was recorded. Participant characteristics and routine laboratory tests were collected for each participant and recorded in an anonymized Excel file. The data of 75 participants from center1, 414 participants from center2, and 110 participants from center3 have already been presented in two published studies conducted for other purposes [8, 9].

### AC measurement

**Fig. 2:**
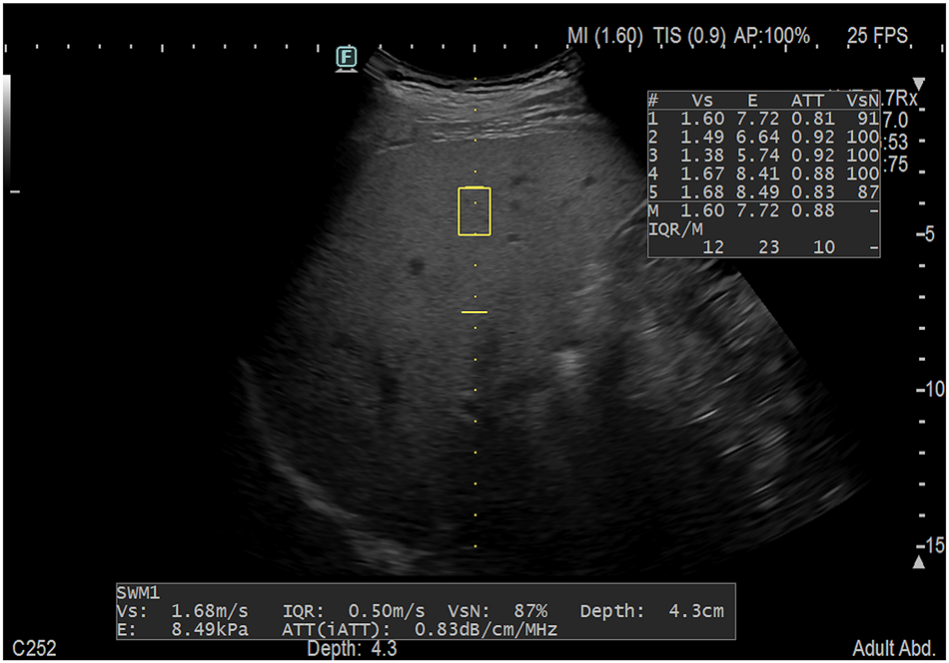
Fifty-year-old man with steatotic liver disease (BMI: 29.9 kg/m^2^; MRI-PDFF: 17.8%). Estimation of liver fat content using the AC implemented in the Arietta 850 US system (iATT, Fujifilm). The commercially available software currently has an ROI length of 40 mm set at a fixed distance from the transducer (from 35 mm to 75 mm). Two horizontal yellow lines indicate the width and length of the AC ROI. The liver stiffness measurement is calculated along the same axis as the AC measurement, but in a different ROI (yellow box). For each acquisition, the system automatically provides the interquartile range/median (IQR/M) for both AC and liver stiffness. Vs, shear wave speed; E, elasticity; ATT (iATT), attenuation coefficient; VsN, manufacturer’s reliability index for the shear wave measurement; M, median; IQR/M, interquartile range/median; SWM, shear wave measurement; m/s, meter/second; kPa, kilopascal; dB/cm/MHz, decibel/centimeter/megahertz

AC measurements were obtained with the iATT algorithm (software level: version 5.x) implemented in the Arietta 850 US system (Fujifilm). The commercially available iATT algorithm calculates the AC in an ROI of fixed size, measuring 40 mm in length, and from 35 mm to 75 mm deep from the transducer. A reference table for depth adjustments is embedded into the algorithm. AC measurements were performed by positioning the transducer (C252 convex array with frequencies from 1 MHz to 6 MHz) in the right intercostal space while the participants held their breath (Fig. 2). All patients were asked to fast for a minimum of 4 h. The US measurements were obtained without knowledge of the MRI-PDFF results.

AC measurements were performed by M.H. who had 25 years of experience in US examination and two other operators, who had 9 years and 16 years of experience, at center1; by S.O. and another operator, both had 15 years of experience, at center2; by Y.Y. and four other operators who had 25, 15, 13, 12, and 7 years of experience, respectively, at center3; and by R.G.B. who had 30 years of experience at center4.

Since the stiffness value is obtained together with the AC value, the protocol recommended by liver elastography guidelines was followed for the acquisition of the AC values [10, 11]. Raw data were automatically collected and stored on the hard drive of the US system. The skin-to-liver capsule distance was measured on a frozen B-mode image. AC values were obtained using the standard commercially available iATT algorithm, i.e., the one with an ROI of fixed length (40 mm) and depth (35–75 mm from the transducer) (ROI-setting1). At least five AC acquisitions were obtained for each participant, and the median value was used for statistical analysis. For this study, Fujifilm engineers reprocessed raw data of all AC acquisitions at the authors’ request. The ROI was shortened to 30 mm and positioned between 45 mm and 75 mm from the transducer (ROI-setting2). This setting was chosen considering that patients with liver steatosis are frequently obese and have thick subcutaneous tissue. Moreover, following the WFUMB guidance for AC measurements [6], a movable ROI with a length of 30 mm was positioned with the top edge at 20 mm under the liver capsule (ROI-setting3) for AC measurements. Additionally, AC measurements were obtained with a movable 30 mm ROI with the top edge at 25 mm under the liver capsule (ROI-setting4). The authors independently conducted the data analysis and manuscript preparation without any influence from Fujifilm.

### MRI-PDFF acquisition

PDFF values were obtained by using the following MRI systems: IDEAL IQ, SIGNA Architect, 3.0 Tesla (GE HealthCare) at center1; Discovery MR750w 3.0 Tesla (GE HealthCare) at center2; Ingenia 1.5 Tesla, (Philips Healthcare) at center3; Galan 3.0 Tesla (Canon Medical Systems) at center4.

Four ROIs were placed in different areas of the liver, avoiding large vessels, focal lesions, artifacts, focal fatty deposition or focal fatty sparing areas, and the liver edge, and the mean value of the four measurements was used for statistical analysis. MRI-PDFF cutoff values for liver steatosis (*S*) > 0, *S* > 1, and *S* > 2, respectively, were 6%, 17.1%, and ≥ 22.1% [12].

MRI-PDFF was performed within 3 months before or after AC measurements in center1, center2, and center3, and on the same day in center4. The participants did not receive any interventions, such as treatment or lifestyle changes, between the AC measurements and the MRI-PDFF procedure. The operators who performed the MRI-PDFF examinations in center2 and center4 were different from those who performed the AC acquisitions, whereas they were the same in center1 and center3.

### Statistical analysis

A required sample size of 745 was obtained through a power analysis based on the area under the receiver operating characteristic curve (AUROC) [13]. The calculation was performed considering an expected AUROC of 0.85, a margin of error of 0.03, a power of 90%, and a significance level of 5%.

The Kolmogorov–Smirnov test assessed the normality of quantitative variables. Continuous variables were expressed as median and interquartile range (IQR) due to their non-normal distribution. Qualitative variables were presented as counts and percentages.

Continuous variables were compared using the Student’s *t*-test or one-way ANOVA for normally distributed data, and the Mann–Whitney *U*-test or Kruskal–Wallis test for non-normally distributed data, with post hoc analyses applied where significant differences were identified. Categorical variables were analyzed using Chi-square or Fisher’s exact test, as appropriate.

The correlation between PDFF and AC was assessed using Spearman’s rank correlation coefficient. This non-parametric method was chosen because neither variable did not followed a normal distribution. Correlation strength was interpreted as negligible (< 0.30), poor (≥ 0.30 and < 0.50), moderate (≥ 0.50 and < 0.70), high (≥ 0.70 and < 0.90), or very high (≥ 0.90) [14].

The diagnostic performance for different grades of steatosis based on AC measurements was assessed using ROC curve analysis, with MRI-PDFF as the reference standard [15]. AUROC was used to evaluate diagnostic performance, with thresholds interpreted as no discrimination (0.50), poor (0.50–0.70), acceptable (0.70–0.80), excellent (0.80–0.90), and outstanding (> 0.90) [16]. AUROCs were compared using DeLong’s test [17]. Statistical significance was defined as *p* < 0.05, with all tests being two-sided.

Analyses were conducted by D.R. using R version 4.2.2 (R Core Team, 2022), employing packages such as gtsummary for summarizing data and creating tables, dplyr for data manipulation, psych for correlations, and pROC for ROC analysis.

## Results

### Participant characteristics

A total of 769 individuals from four different centers were enrolled. Nineteen patients from center2 were excluded due to contraindications for performing MRI-PDFF. Seven hundred fifty individuals (366 females; median age: 65 years, IQR: 52; 73) met the inclusion criteria. The prevalence of liver steatosis (*S* > 0), as assessed by MRI-PDFF, was 46.4%. The baseline characteristics of the overall study population and stratified by center are presented in Table 1. Overall, for four different ROI settings, a total of 3000 observations were collected from 750 participants.

**Table 1:** Study cohort characteristics, overall and by center

Variable	Overall	Center1	Center2	Center3	Center4	*p* value^b^
	*N* = 750^a^	*N* = 144^a^	*N* = 446^a^	*N* = 110^a^	*N* = 50^a^	
Age (years)	65 (52, 73)	62 (50, 71)	65 (55, 75)	67 (50, 73)	55 (42, 62)	< 0.001
Gender						< 0.001
Female	366 (49%)	93 (65%)	183 (41%)	55 (50%)	35 (70%)	
Male	384 (51%)	51 (35%)	263 (59%)	55 (50%)	15 (30%)	
Etiology						< 0.001
Alcohol	22 (3%)	9 (6%)	0 (0%)	12 (11%)	1 (2%)	
Autoimmune	52 (7%)	44 (31%)	0 (0%)	8 (7%)	0 (0%)	
MASLD/MASH	307 (41%)	62 (43%)	160 (36%)	36 (33%)	49 (98%)	
Other	126 (17%)	7 (5%)	102 (23%)	17 (15%)	0 (0%)	
Viral	238 (32%)	22 (15%)	179 (41%)	37 (34%)	0 (0%)	
BMI (kg/m^2^)	24.8 (22.1, 28.5)	25.8 (22, 30.7)	24.5 (21.8, 27.1)	24.2 (22.3, 27.6)	32.4 (28.3, 38)	< 0.001
Obesity^c^	206 (27%)	55 (38%)	90 (20%)	26 (24%)	35 (70%)	< 0.001
SCD (mm)	18 (15, 21)	18.9 (15, 22.9)	17 (14, 20)	18 (15, 20)	24 (21, 31)	< 0.001
SCD > 25 mm	85 (11%)	24 (17%)	30 (7%)	7 (6%)	24 (48%)	< 0.001
ALT (UI/L)	28 (17, 52)	34 (21, 56)	28 (17, 55)	27 (18, 44)	21 (16, 35)	0.004
AST (UI/L)	28 (21, 47)	35 (24, 52)	27 (21, 49)	29 (23, 43)	20 (16, 28)	< 0.001
Platelets	192 (128, 242)	187 (112, 252)	188 (124, 235)	167 (111, 213)	258 (238, 289)	< 0.001
Diabetes	156 (24%)	12 (29%)	98 (22%)	22 (20%)	24 (48%)	< 0.001
FIB-4	1.74 (1.07, 3.17)	1.47 (0.86, 3.09)	1.93 (1.19, 3.29)	2.16 (1.31, 3.33)	0.88 (0.62, 1.27)	0.01
FIB-4 categories						< 0.001
FIB-4 < 1.3	239 (34%)	47 (46%)	127 (29%)	26 (24%)	39 (78%)	
FIB-4 > 2.67	224 (34%)	31 (30%)	151 (34%)	42 (38%)	0 (0%)	
1.3 < FIB-4 < 2.67	243 (32%)	25 (24%)	165 (37%)	42 (38%)	11 (22%)	
MRI-PDFF (%)	5.3 (2.5, 11.9)	3.8 (2.2, 9.9)	4.9 (2.3, 11)	6.2 (3.2, 13.1)	9.6 (5.1, 18.9)	< 0.001
Steatosis > 0	348 (46%)	55 (38%)	199 (45%)	58 (53%)	36 (72%)	< 0.001
Steatosis > 1	109 (15%)	17 (12%)	54 (12%)	22 (20%)	16 (32%)	< 0.001
Steatosis > 2	66 (9%)	12 (8%)	32 (7%)	12 (11%)	10 (20%)	0.02
Steatosis categories						< 0.001
No steatosis	402 (54%)	89 (62%)	247 (55%)	52 (47%)	14 (28%)	
Mild	239 (32%)	38 (26%)	145 (33%)	36 (33%)	20 (40%)	
Moderate	43 (6%)	5 (4%)	22 (5%)	10 (9%)	6 (12%)	
Severe	66 (9%)	12 (8%)	32 (7%)	12 (11%)	10 (20%)	
AC ROI-setting1 (dB/cm/MHz)	0.68 (0.59, 0.78)	0.67 (0.59, 0.76)	0.66 (0.58, 0.77)	0.70 (0.62, 0.79)	0.78 (0.70, 0.86)	< 0.001
AC ROI-setting2 (dB/cm/MHz)	0.63 (0.53, 0.76)	0.62 (0.55, 0.74)	0.62 (0.52, 0.76)	0.65 (0.56, 0.75)	0.75 (0.64, 0.85)	< 0.001
AC ROI-setting3 (dB/cm/MHz)	0.63 (0.52, 0.77)	0.62 (0.55, 0.75)	0.60 (0.50, 0.75)	0.65 (0.54, 0.77)	0.80 (0.71, 0.91)	< 0.001
AC-ROI-setting4 (dB/cm/MHz)	0.68 (0.56, 0.82)	0.66 (0.58, 0.78)	0.66 (0.54, 0.81)	0.70 (0.58, 0.81)	0.81 (0.72, 0.89)	< 0.001

*AST* aspartate aminotransferase, *ALT* alanine aminotransferase, *BMI* body mass index, *FIB-4* fibrosis 4 score, *MASLD/MASH* metabolic dysfunction-associated steatotic liver disease/metabolic dysfunction-associated steatohepatitis, *MRI-PDFF* magnetic resonance imaging proton density fat fraction, *ROI* region of interest, *SCD* skin-to-liver capsule distance

^a^ Median (IQR) or number (%)

^b^ Kruskal–Wallis rank sum test; Pearson’s Chi-squared test; Fisher’s exact test

^c^ BMI ≥ 30 for North American participants and BMI ≥ 28 for Japanese participants

### AC values

The median (IQR) AC values for S = 0, S > 0, S > 1, and S > 2 were 0.56 (0.50, 0.63), 0.78 (0.70, 0.85), 0.83 (0.77, 0.91), and 0.85 (0.78, 0.92) dB/cm/MHz, respectively (*p* < 0.001).

The median (IQR) AC values for the different ROI settings are reported in Table 1.

The correlation between AC values and MRI-PDFF was high across different ROI settings for the overall population and for subgroups with skin-to-liver capsule distance ≤ 25 mm and non-obese participants, with ROI-setting4 showing the strongest correlation in all groups. In obese participants and those with a skin-to-liver capsule distance > 25 mm, the correlation weakened, ranging from poor to moderate and from negligible to poor, respectively, with ROI-setting4 still showing the best correlation.

When analyzed by the enrolling center, the correlation was worse for centers with a high proportion of obese participants and participants with skin-to-liver capsule distance > 25 mm, such as center1 and center4, which also had the highest prevalence of participants with MASLD.

When obesity was combined with skin-to-liver capsule distance ≤ 25 mm, the correlation improved compared to that of obesity alone, while combining obesity with skin-to-liver capsule distance > 25 mm, the correlation remained consistent with that of skin-to-liver capsule distance > 25 mm. Notably, the ROI-setting4 consistently showed the best correlation across all subgroups (Table 2). The electronic supplementary material presents a graphical visualization of the correlations.

**Table 2:** Correlation between MRI-PDFF and ultrasound AC measured at different ROI settings using Spearman’s rank correlation coefficient (Spearman’s rho) in the overall population and in the different subgroups according to skin-to-liver capsule distance, BMI, and center

	ROI-setting	Spearman’s rho (95% CI)	*p* value
Overall (*n* = 750)	1	0.74 (0.72; 0.78)	< 0.001
	2	0.76 (0.73; 0.79)	< 0.001
	3	0.76 (0.73; 0.79)	< 0.001
	4	0.79 (0.77; 0.82)	< 0.001
SCD ≤ 25 mm (*n* = 686)	1	0.76 (0.73; 0.79)	< 0.001
	2	0.76 (0.73; 0.79)	< 0.001
	3	0.76 (0.73; 0.79)	< 0.001
	4	0.79 (0.77; 0.82)	< 0.001
SCD > 25 mm (*n* = 64)	1	0.18 (−0.11; 0.43)	< 0.001
	2	0.39 (0.16; 0.58)	< 0.001
	3	0.44 (0.19; 0.63)	< 0.001
	4	0.47 (0.24; 0.66)	< 0.001
No obesity^a^ (*n* = 544)	1	0.74 (0.70; 0.78)	< 0.001
	2	0.72 (0.70; 0.76)	< 0.001
	3	0.73 (0.69; 0.77)	< 0.001
	4	0.76 (0.71; 0.79)	< 0.001
Obesity^a^ (*n* = 206)	1	0.46 (0.34; 0.58)	< 0.001
	2	0.59 (0.49; 0.68)	< 0.001
	3	0.57 (0.46; 0.66)	< 0.001
	4	0.66 (0.57; 0.73)	< 0.001
Obesity^a^ and SCD ≤ 25 mm (*n* = 143)	1	0.58 (0.45; 0.70)	< 0.001
	2	0.68 (0.57; 0.77)	< 0.001
	3	0.66 (0.55; 0.75)	< 0.001
	4	0.75 (0.66; 0.82)	< 0.001
Obesity^a^ and SCD > 25 mm (*n* = 63)	1	0.17 (−0.13; 0.44)	< 0.001
	2	0.38 (0.12; 0.58)	< 0.001
	3	0.43 (0.20; 0.63)	< 0.001
	4	0.46 (0.22; 0.67)	< 0.001
Center1 (*n* = 144)	1	0.60 (0.50; 0.69)	< 0.001
	2	0.59 (0.48; 0.68)	< 0.001
	3	0.64 (0.53; 0.72)	< 0.001
	4	0.67 (0.58; 0.75)	< 0.001
Center2 (*n* = 446)	1	0.80 (0.77; 0.83)	< 0.001
	2	0.80 (0.77; 0.83)	< 0.001
	3	0.80 (0.77; 0.83)	< 0.001
	4	0.82 (0.80; 0.85)	< 0.001
Center3 (*n* = 110)	1	0.70 (0.59; 0.78)	< 0.001
	2	0.73 (0.64; 0.81)	< 0.001
	3	0.75 (0.66; 0.81)	< 0.001
	4	0.80 (0.71; 0.85)	< 0.001
Center4 (*n* = 50)	1	0.46 (0.21; 0.68)	< 0.001
	2	0.71 (0.55; 0.82)	< 0.001
	3	0.74 (0.57; 0.84)	< 0.001
	4	0.80 (0.65; 0.87)	< 0.001

*ROI* region of interest, *SCD* skin-to-liver capsule distance

^a^ BMI ≥ 30 for North American participants and BMI ≥ 28 for Japanese participants

### Diagnostic performance

**Fig. 3:**
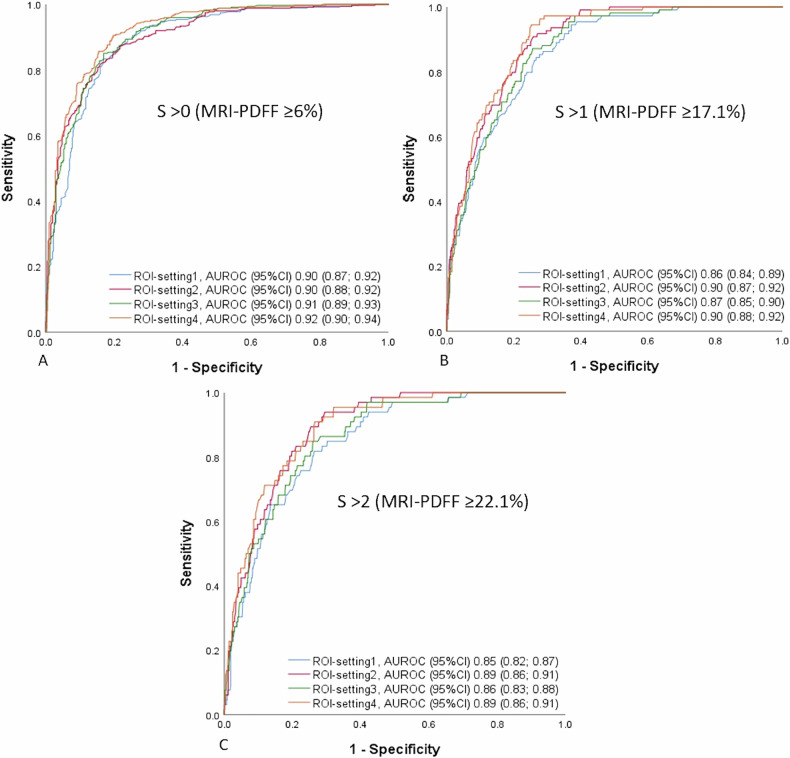
Diagnostic performance of the ultrasound AC measured at different ROI settings for detection of steatosis grade > 0 (**A**), > 1 (**B**), and > 2 (**C**) using MRI-PDFF as reference standard in the overall population

The diagnostic performance of AC values across different ROI settings in the overall population was outstanding for *S* > 0, ranged from excellent to outstanding for *S* > 1, and was excellent for *S* > 2 (Fig. 3).

In the subgroups with skin-to-liver capsule distance ≤ 25 mm and of non-obese participants, the performance ranged from excellent to outstanding across all grades of liver steatosis.

In obese participants and those with the skin-to-liver capsule distance > 25 mm, the performance weakened across all grades of liver steatosis, ranging from acceptable to excellent and from poor to acceptable, respectively.

In the analysis according to the enrolling center, the performance was lower for centers with a higher proportion of participants with obesity or with the skin-to-liver capsule distance > 25 mm, such as center1 and center4.

The performance was significantly better across all ROI settings in cases with a skin-to-liver capsule distance ≤ 25 mm compared to those with a skin-to-liver capsule distance > 25 mm, while in non-obese participants, ROI-setting1 and ROI-setting2 performed better compared to those of obese subjects (Table 3).

**Table 3:** Diagnostic performance of ultrasound AC measured at different ROI settings for detecting different grades of steatosis using MRI-PDFF as reference standard in the overall population and in the different subgroups according to skin-to-liver capsule distance, BMI, and center

	ROI-setting	*S* > 0 (MRI-PDFF ≥ 6%)	*S* > 1 (MRI-PDFF ≥ 17.1%)	*S* > 2 (MRI-PDFF ≥ 22.1%)
		AUROC (95% CI)	AUROC (95% CI)	AUROC (95% CI)
Overall (*n* = 750)	1	0.90 (0.87; 0.92)	0.86 (0.84; 0.89)	0.85 (0.82; 0.87)
	2	0.90 (0.88; 0.92)	0.90 (0.87; 0.92)***	0.89 (0.86; 0.91)**
	3	0.91 (0.89; 0.93)*	0.87 (0.85; 0.90)	0.86 (0.83; 0.88)
	4	0.92 (0.90; 0.94)***	0.90 (0.88; 0.92)**	0.89 (0.86; 0.91)*
SCD ≤ 25 mm (*n* = 686)	1	0.91 (0.89; 0.93)	0.89 (0.86; 0.91)	0.88 (0.84; 0.91)
	2	0.91 (0.89; 0.93)	0.91 (0.88; 0.93)	0.90 (0.87; 0.93)
	3	0.91 (0.89; 0.93)	0.88 (0.85; 0.92)	0.86 (0.82; 0.91)
	4	0.93 (0.91; 0.95)**	0.91 (0.89; 0.94)	0.90 (0.86; 0.93)
SCD > 25 mm (*n* = 64)	1	0.54 (0.40; 0.69)^†††^	0.67 (0.50; 0.84)	0.60 (0.41; 0.78)
	2	0.68 (0.54; 0.82)^††^	0.75 (0.62; 0.89)	0.70 (0.54; 0.85)
	3	0.71 (0.54; 0.88)*,^†^	0.76 (0.64; 0.89)	0.75 (0.61; 0.88)
	4	0.72 (0.55; 0.89)*,^†^	0.77 (0.64; 0.90)	0.75 (0.61; 0.90)
No obesity^a^ (*n* = 544)	1	0.91 (0.88; 0.93)^†††^	0.91 (0.88; 0.94)	0.91 (0.87; 0.95)
	2	0.90 (0.87; 0.93)^†^	0.92 (0.90; 0.95)	0.92 (0.89; 0.96)
	3	0.90 (0.88; 0.93)	0.88 (0.84; 0.92)	0.85 (0.79; 0.92)
	4	0.92 (0.90; 0.94)*	0.91 (0.88; 0.94)	0.89 (0.84; 0.94)
Obesity^a^ (*n* = 206)	1	0.75 (0.68; 0.83)	0.75 (0.67; 0.82)	0.70 (0.60; 0.79)
	2	0.82 (0.75; 0.89)*	0.81 (0.74; 0.87)	0.78 (0.70; 0.85)
	3	0.82 (0.74; 0.89)	0.79 (0.71; 0.85)	0.78 (0.71; 0.85)
	4	0.85 (0.79; 0.92)*	0.84 (0.79; 0.90)	0.83 (0.77; 0.90)
Obesity^a^ and SCD ≤ 25 mm (*n* = 143)	1	0.83 (0.75; 0.92)^†††^	0.79 (0.71; 0.86)	0.75 (0.65; 0.84)
	2	0.87 (0.80; 0.94)^††^	0.84 (0.77; 0.90)	0.81 (0.73; 0.89)
	3	0.87 (0.81; 0.94)^†^	0.84 (0.77; 0.90)	0.80 (0.72; 0.88)
	4	0.91 (0.85; 0.96)*,^†^	0.89 (0.84; 0.94)	0.86 (0.79; 0.93)
Obesity^a^ and SCD > 25 mm (*n* = 63)	1	0.53 (0.39; 0.68)	0.65 (0.48; 0.83)	0.61 (0.42; 0.80)
	2	0.67 (0.53; 0.81)	0.74 (0.60; 0.89)	0.71 (0.55; 0.86)
	3	0.70 (0.53; 0.88)	0.76 (0.63; 0.88)	0.75 (0.62; 0.89)
	4	0.72 (0.55; 0.89)*	0.76 (0.63; 0.90)	0.76 (0.62; 0.90)
Center1 (*n* = 144)	1	0.82 (0.76; 0.89)	0.84 (0.74; 0.93)	0.85 (0.76; 0.94)
	2	0.82 (0.75; 0.89)	0.87 (0.79; 0.95)	0.87 (0.78; 0.97)
	3	0.85 (0.78; 0.91)	0.80 (0.68; 0.91)	0.74 (0.59; 0.89)
	4	0.87 (0.81; 0.92)	0.85 (0.76; 0.93)	0.82 (0.70; 0.93)
Center2 (*n* = 446)	1	0.93 (0.91; 0.96)	0.89 (0.86; 0.93)	0.89 (0.85; 0.93)
	2	0.93 (0.91; 0.96)	0.91 (0.88; 0.94)	0.92 (0.88; 0.95)
	3	0.93 (0.91; 0.96)	0.89 (0.86; 0.92)	0.88 (0.83; 0.93)
	4	0.94 (0.92; 0.96)	0.91 (0.88; 0.94)	0.91 (0.87; 0.95)
Center3 (*n* = 110)	1	0.86 (0.79; 0.93)	0.83 (0.74; 0.92)	0.80 (0.66; 0.94)
	2	0.86 (0.79; 0.93)	0.90 (0.85; 0.96)	0.88 (0.80; 0.96)
	3	0.89 (0.83; 0.95)	0.87 (0.80; 0.95)	0.91 (0.84; 0.98)
	4	0.91 (0.85; 0.96)	0.93 (0.88; 0.98)	0.93 (0.88; 0.99)
Center4 (*n* = 50)	1	0.68 (0.52; 0.84)	0.75 (0.59; 0.91)	0.62 (0.40; 0.84)
	2	0.84 (0.73; 0.96)**	0.84 (0.73; 0.96)	0.74 (0.59; 0.90)
	3	0.87 (0.77; 0.97)***	0.85 (0.75; 0.96)	0.80 (0.67; 0.94)
	4	0.91 (0.83; 0.99)***	0.88 (0.78; 0.97)	0.82 (0.69; 0.95)

*ROI* region of interest, *SCD* skin-to-liver capsule distance

^a^ BMI ≥ 30 for North American participants and BMI ≥ 28 for Japanese participants

Comparison of ROI-setting1 with other ROI settings within the same subgroup: *p* < 0.05*, *p* < 0.01**, *p* < 0.001***

Comparison of the same ROI-setting between subgroups (obesity vs no obesity; obesity and SLC distance ≤ 25 mm vs obesity and SLC distance > 25 mm): *p* < 0.05^†^, *p* < 0.01^††^, *p* < 0.001^†††^

Performance was evaluated using ROC curve analysis and expressed by the AUROC. The comparison between the ROI setting1 and other ROI settings was performed with the DeLong test for all grades of liver steatosis in the overall population and for *S* > 0 in all subgroups

When obesity was combined with a skin-to-liver capsule distance ≤ 25 mm, the performance improved compared to that of obesity alone, while combining obesity with a skin-to-liver capsule distance > 25 mm, the performance remained consistent with that of a skin-to-liver capsule distance > 25 mm (Table 3).

**Fig. 4:**
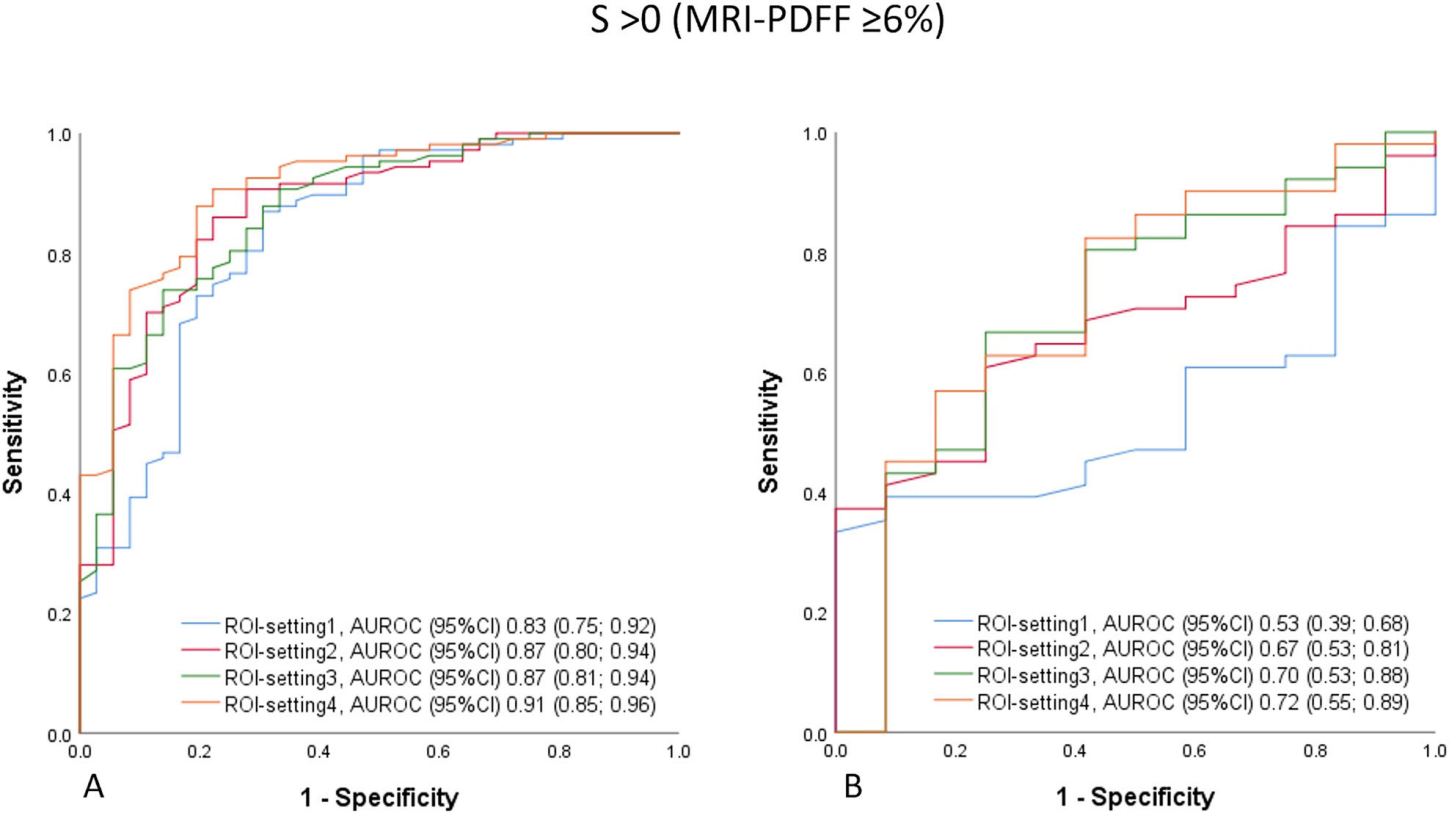
Diagnostic performance of the ultrasound AC measured at different ROI settings for detection of steatosis grade > 0 using MRI-PDFF as reference standard in the subgroup of obese* subjects with skin-to-capsule distance ≤ 25 mm (**A**) and > 25 mm (**B**). * BMI ≥ 30 for North American participants and BMI ≥ 28 for Japanese participants

The ROI-setting4 showed significantly the best performance across all grades of liver steatosis in the overall population. For detecting steatosis (*S* > 0), the ROI-setting4 constantly performed significantly better than ROI-setting1 in participants with obesity and skin-to-liver distance > 25 mm (Table 3 and Fig. 4).

## Discussion

The results of this study showed that (i) the correlation of the AC with MRI-PDFF increased with a movable ROI, and this finding was more pronounced in individuals with obesity and in those with thick subcutaneous tissue. i.e., higher than 25 mm; (ii) the highest performance of the AC for all grades of liver steatosis was obtained with the upper edge of the ROI positioned at 25 mm below the liver capsule; and (iii) using a fixed ROI setting in individuals with a skin-to-liver capsule distance greater than 25 mm, AC performance for detecting steatosis (*S* > 0) significantly decreased.

These results have important implications for the proper use of AC algorithms in general. Indeed, some vendors have established a fixed distance from the transducer of the ROI position with the objective of mitigating the discrepancies in AC values when the user is permitted to adjust the ROI position [7, 18]. However, this approach fails to consider the fact that the thickness of the subcutaneous tissue varies among individuals. With this setting, the ROI can be very close to the liver capsule in individuals with thick subcutaneous tissue. Therefore, reverberation artefacts occurring in this area might be included in the ultrasound AC estimate, resulting in the lower performance observed in this study.

The strengths of our study are twofold: first, it includes different cohorts with different body habitus; and second, it evaluates the correlation between the AC and the MRI-PDFF and the performance of the AC using four different settings of the ROI. To the best of our knowledge, this is the first study to evaluate the effect of a fixed position of the ROI vs a freely adjustable position of the ROI on the performance of the AC.

It is noteworthy that the thickness of the subcutaneous tissue had a greater impact on the performance of the AC than did BMI. In fact, for the detection of liver steatosis (*S* > 0) in individuals with a skin-to-liver capsule distance greater than 25 mm, the strength of the correlation with MRI-PDFF was negligible, and the AUROC was poor when using the commercially available algorithm that calculates the AC at a fixed distance of 35–75 mm from the transducer.

It should also be noted that the greatest improvement in performance with the movable ROI at 25 mm below the liver capsule was observed in the North American cohort, which also had the highest thickness of the subcutaneous tissue.

Using AC algorithms with a fixed ROI depth from other vendors, it has been reported that the performance decreases at higher BMI (≥ 30 kg/m^2^), but it was not evaluated whether this finding was predominantly attributable to an increased thickness of the subcutaneous tissue [9, 19, 20]. The results of this study show that the thickness of the subcutaneous tissue is the main factor affecting the performance of the AC algorithm, while BMI plays a minimal role. On the other hand, subjects with obesity are also those who are more likely to have higher skin-to-liver capsule distance, so this effect must be considered when interpreting the results. In addition, efforts should be made by manufacturers to mitigate this limitation.

The recent WFUMB guidance for ultrasound liver fat quantification recommends performing AC measurements by positioning the upper part of the ROI 20 mm below the liver capsule [6]. This recommendation was aimed at mitigating differences between AC values obtained with different algorithms. The results of this study show that there is an improvement in the correlation with MRI-PDFF and performance with a freely adjustable ROI positioned at 25 mm. Further studies are necessary to validate this finding with other vendors’ algorithms.

This study has limitations. First, we used only one vendor’s algorithm, so it remains to be determined whether the results of this study are applicable to other algorithms that calculate AC in an ROI positioned at a fixed distance from the transducer. Second, the number of participants with a skin-to-liver capsule distance greater than 25 mm was small, so this finding needs to be validated in larger series. Third, due to the limited number of participants with significant and severe steatosis, a comparison between the standard ROI setting for grading liver steatosis and all other ROI settings in the various subgroups was not feasible. Fourth, we did not use quality measures, such as the IQR-to-median ratio, to evaluate the reliability of AC measurements. Fifth, the results may not be applicable to Western populations because most of the participants were Asian. However, given that the observed differences were driven primarily by BMI or subcutaneous tissue thickness, an analysis was conducted for these two variables. Fifth, inter-observer variability was not evaluated; nevertheless, the use of identical radiofrequency data in the assessment ensures consistency across the results.

In conclusion, a movable ROI improves both the correlation between the AC and MRI-PDFF, as well as the performance of the AC. The highest improvement was observed when the ROI was positioned at 25 mm beneath the liver capsule.

## Supplementary information

ELECTRONIC SUPPLEMENTARY MATERIAL

## Abbreviations

AC: Attenuation coefficient
AUROC: Area under the ROC curve
BMI: Body mass index
IQR: Interquartile range
MASH: Metabolic dysfunction-associated steatohepatitis
MRI-PDFF: Magnetic resonance imaging-derived proton density fat fraction
ROC: Receiver operating characteristic (curve)
ROI: Region of interest
US: Ultrasound
WFUMB: World Federation for Ultrasound in Medicine and Biology
