# Supplementary material for: Optimization of measurements with an ultrasound attenuation coefficient algorithm for quantifying liver fat
Source: Eur Radiol. 2025 Sep 5;36(3):1853–63. doi: 10.1007/s00330-025-11906-5 (PMC12963258; doi:10.1007/s00330-025-11906-5)
Supplement: Supplementary file 1 — ELECTRONIC SUPPLEMENTARY MATERIAL [file 330_2025_11906_MOESM1_ESM.pdf]

Optimization of measurements with an ultrasound attenuation coefficient algorithm for quantifying liver fat

ELECTRONIC SUPPLEMENTARY MATERIAL

The electronic supplementary material presents a graphical visualization of the correlations and is composed of 11 panels, each corresponding to a different analyzed group, including the overall cohort (A), SCD ≤25 mm (B), SCD >25 mm (C), no obesity (D), obesity[a] (E), obesity[a] and SCD ≤25 mm (F), obesity[a] and SCD >25 mm (G), Center 1 (H), Center 2 (I), Center 3 (J) and Center 4 (K).
Within each panel, there are four scatter plots, each representing a different ROI-setting.
In all scatter plots, the x-axis displays MRI-PDFF values, while the y-axis shows the attenuation coefficient values specific to the ROI-setting depicted in each plot. Each black dot represents an individual patient. A continuous black regression line is displayed to illustrate the relationship between MRI-PDFF and attenuation coefficient, and a surrounding grey-shaded area denotes the 95% confidence interval of the regression.
This arrangement enables visual comparison of the association between MRI-PDFF and attenuation coefficients values across multiple ROI-settings and clinical subgroups.

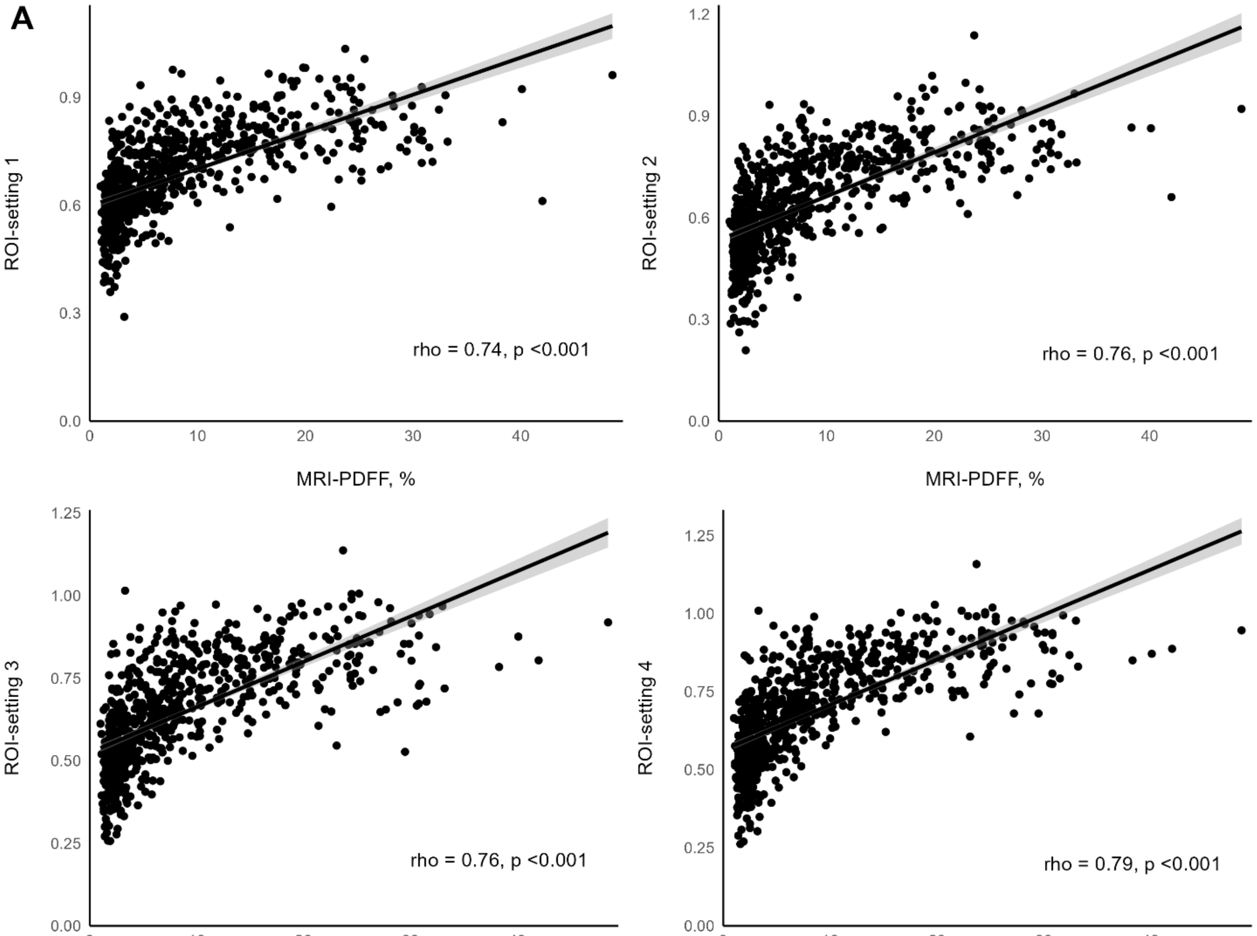

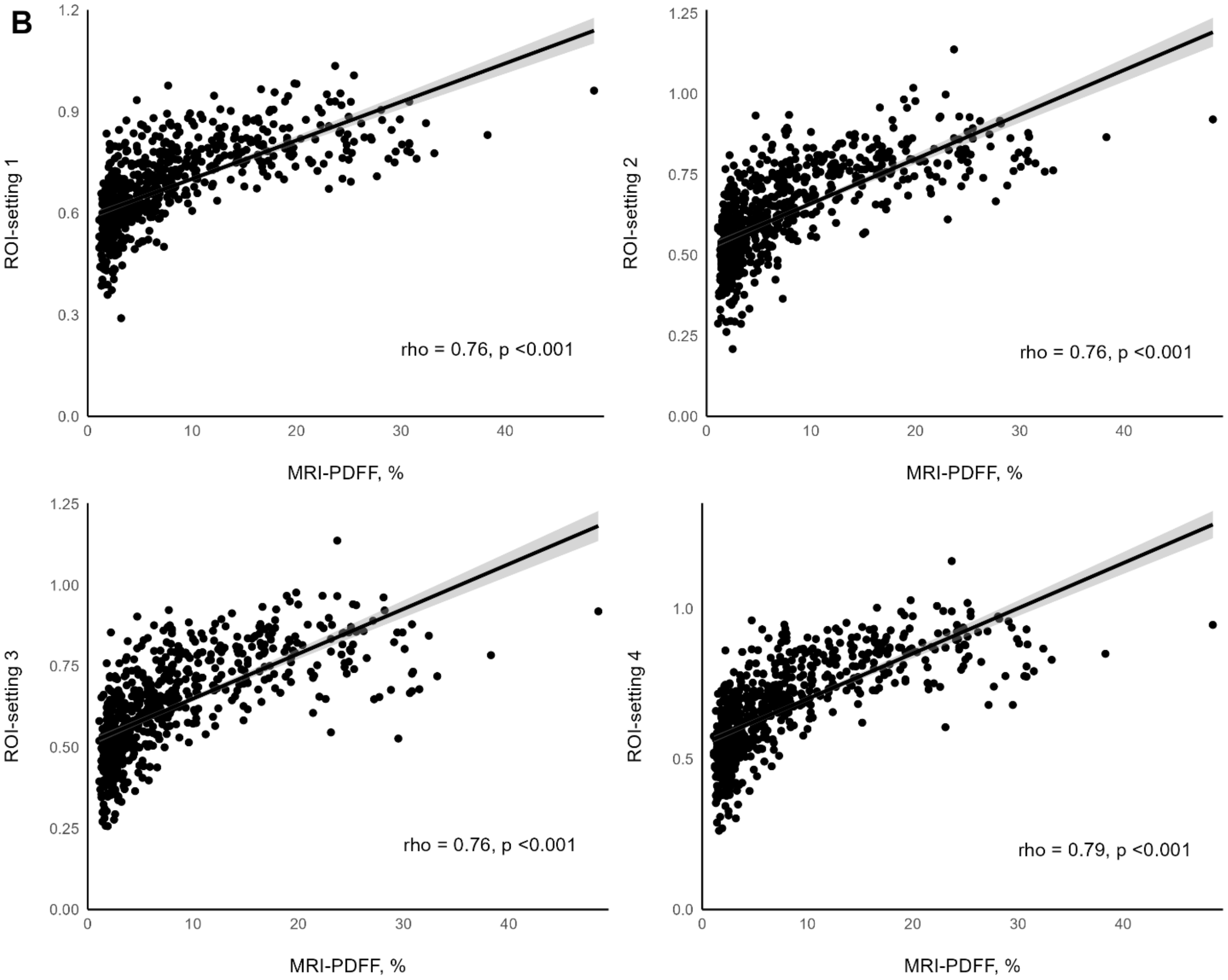
B
ROI-setting 1
rho = 0.76, p <0.001
MRI-PDFF, %
ROI-setting 2
rho = 0.76, p <0.001
MRI-PDFF, %
ROI-setting 3
rho = 0.76, p <0.001
MRI-PDFF, %
ROI-setting 4
rho = 0.79, p <0.001
MRI-PDFF, %

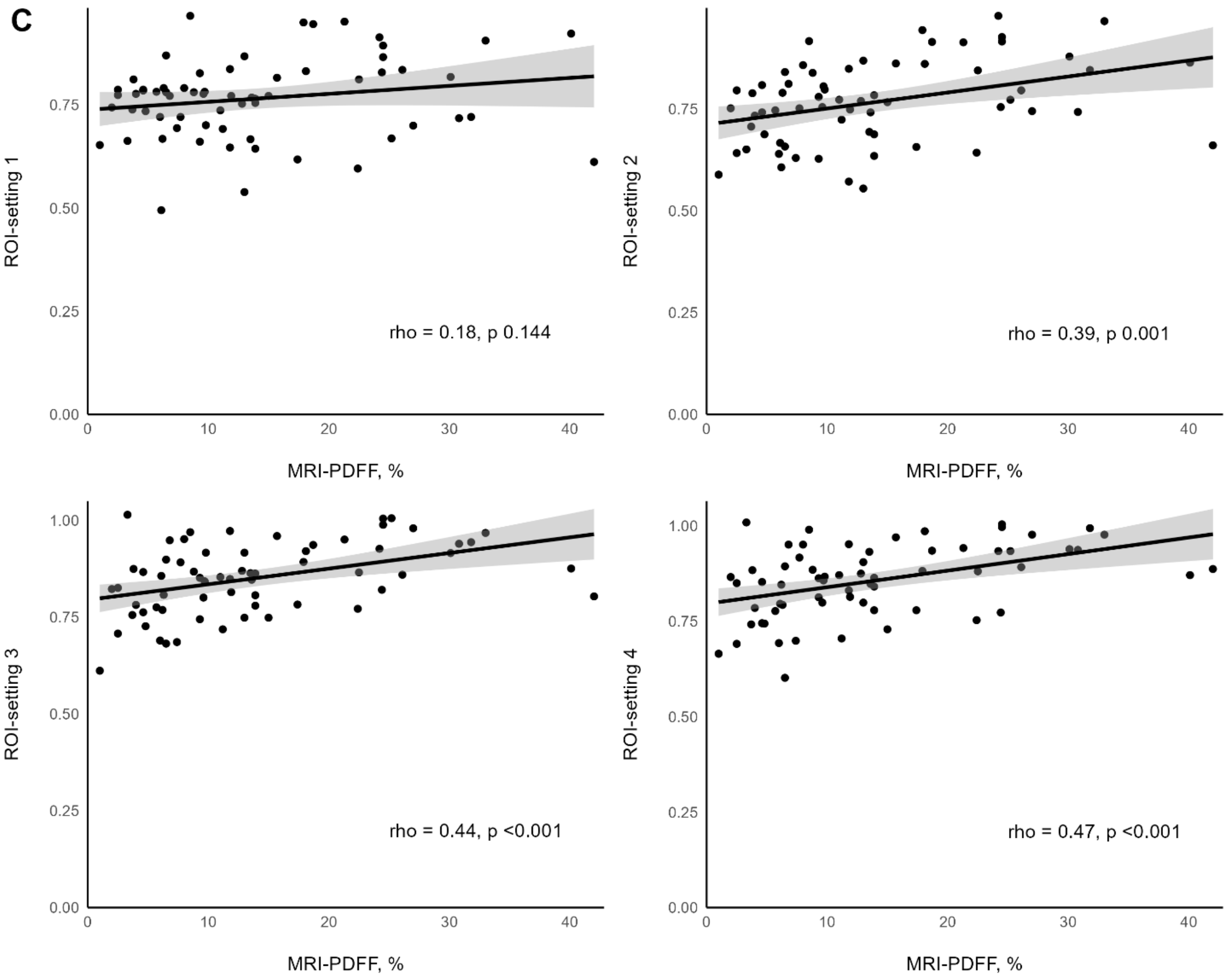

Eur Radiol (2025) Ferraioli G, Roccarina D, Kumada T, et al.

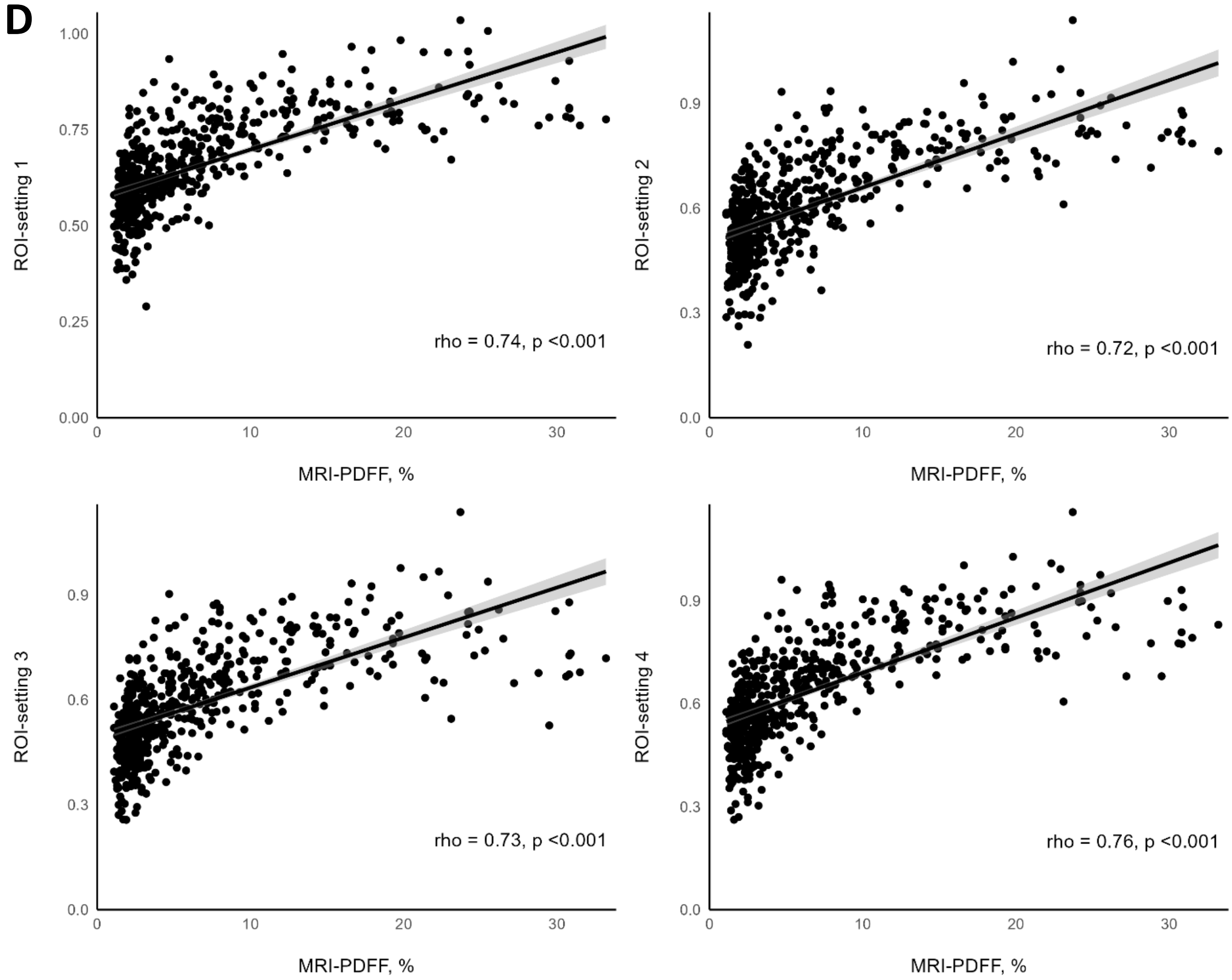
D
ROI-setting 1
rho = 0.74, p <0.001
MRI-PDFF, %
ROI-setting 2
rho = 0.72, p <0.001
MRI-PDFF, %
ROI-setting 3
rho = 0.73, p <0.001
MRI-PDFF, %
ROI-setting 4
rho = 0.76, p <0.001
MRI-PDFF, %

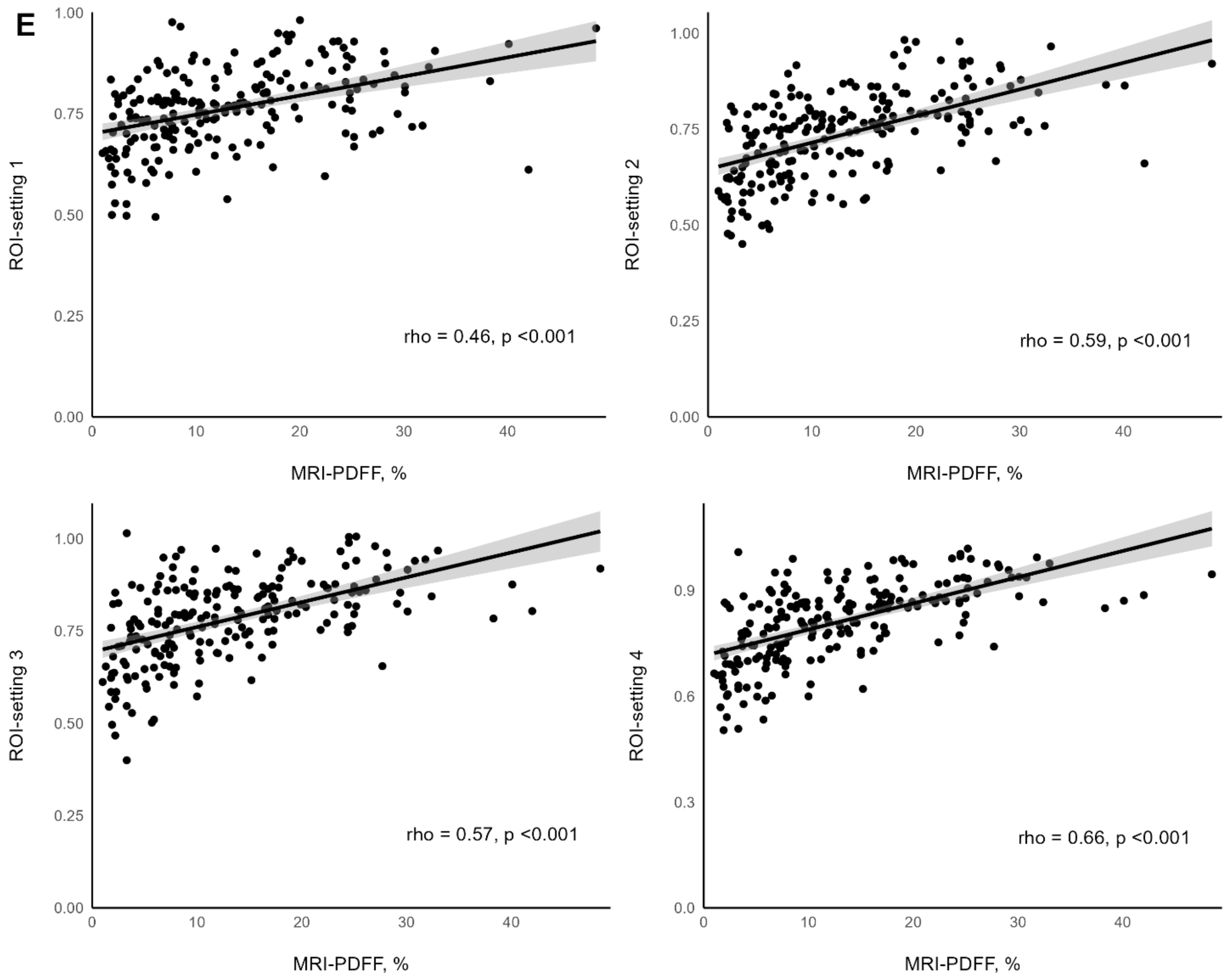
E
ROI-setting 1
MRI-PDFF, %
rho = 0.46, p <0.001
ROI-setting 2
MRI-PDFF, %
rho = 0.59, p <0.001
ROI-setting 3
MRI-PDFF, %
rho = 0.57, p <0.001
ROI-setting 4
MRI-PDFF, %
rho = 0.66, p <0.001

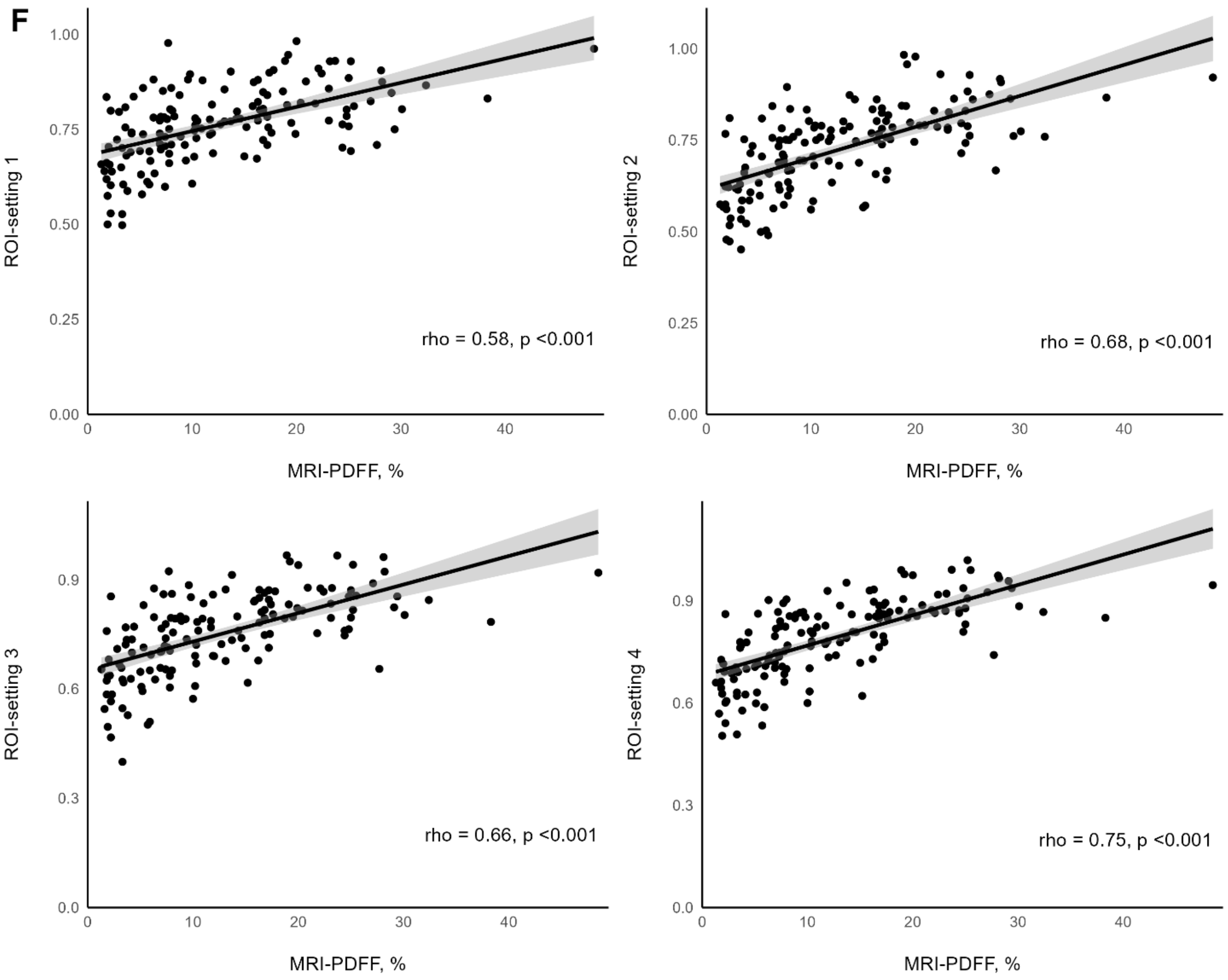

F
ROI-setting 1
rho = 0.58, p <0.001
MRI-PDFF, %
ROI-setting 2
rho = 0.68, p <0.001
MRI-PDFF, %
ROI-setting 3
rho = 0.66, p <0.001
MRI-PDFF, %
ROI-setting 4
rho = 0.75, p <0.001
MRI-PDFF, %

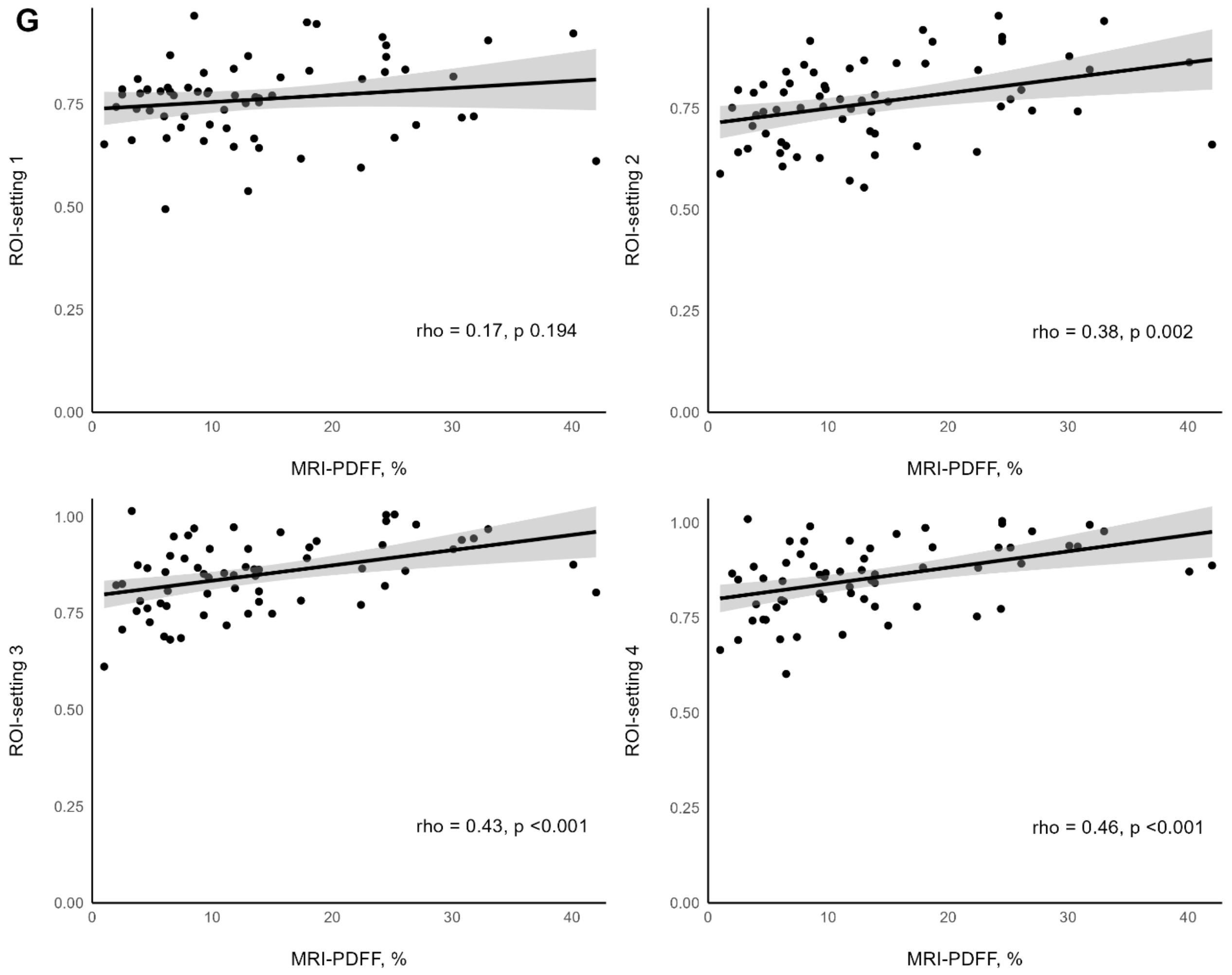
G
ROI-setting 1
rho = 0.17, p 0.194
ROI-setting 2
rho = 0.38, p 0.002
ROI-setting 3
rho = 0.43, p <0.001
ROI-setting 4
rho = 0.46, p <0.001
MRI-PDFF, %

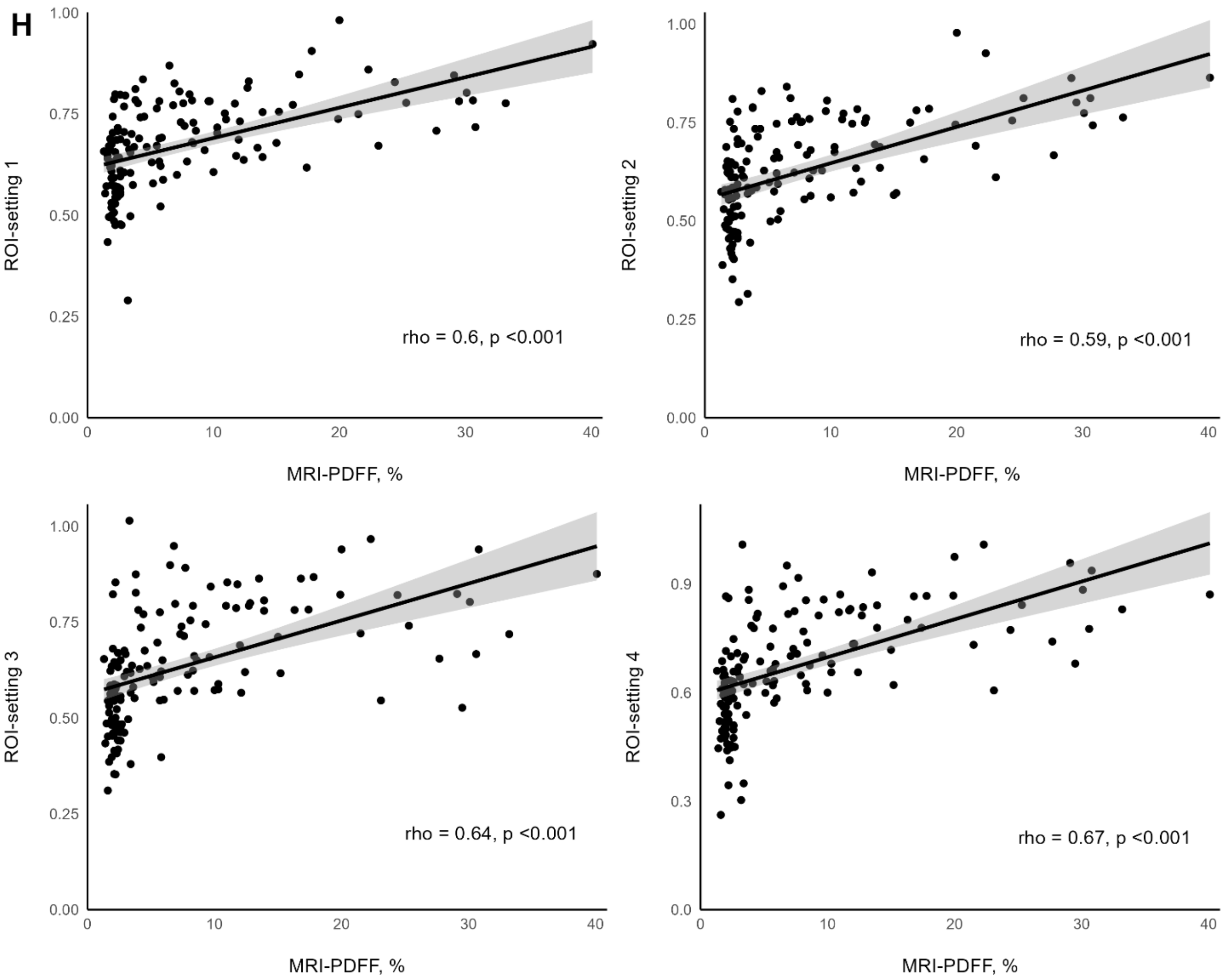

H
ROI-setting 1
rho = 0.6, p <0.001
MRI-PDFF, %
ROI-setting 2
rho = 0.59, p <0.001
MRI-PDFF, %
ROI-setting 3
rho = 0.64, p <0.001
MRI-PDFF, %
ROI-setting 4
rho = 0.67, p <0.001
MRI-PDFF, %

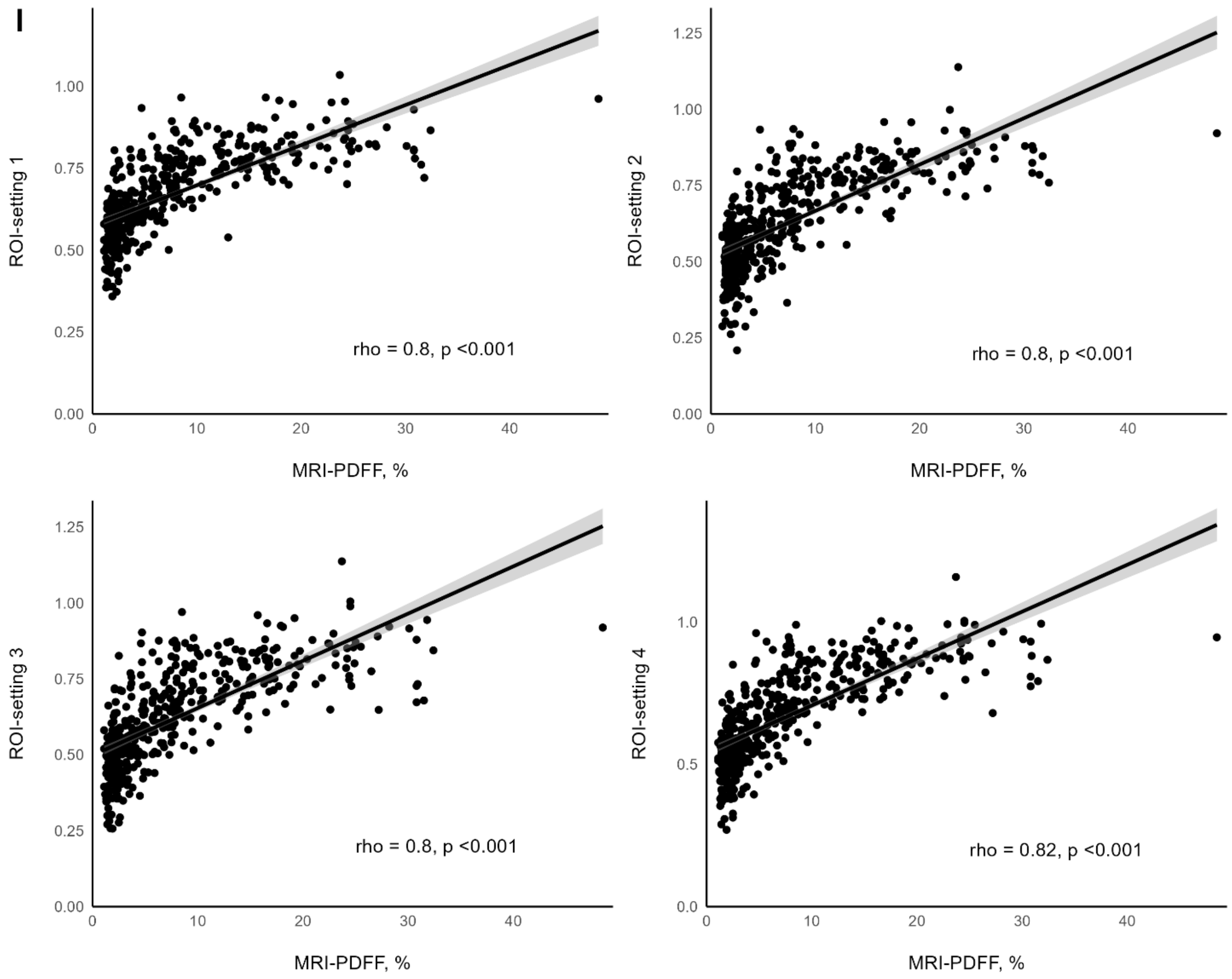
I
ROI-setting 1
rho = 0.8, p <0.001
ROI-setting 2
rho = 0.8, p <0.001
ROI-setting 3
rho = 0.8, p <0.001
ROI-setting 4
rho = 0.82, p <0.001
MRI-PDFF, %

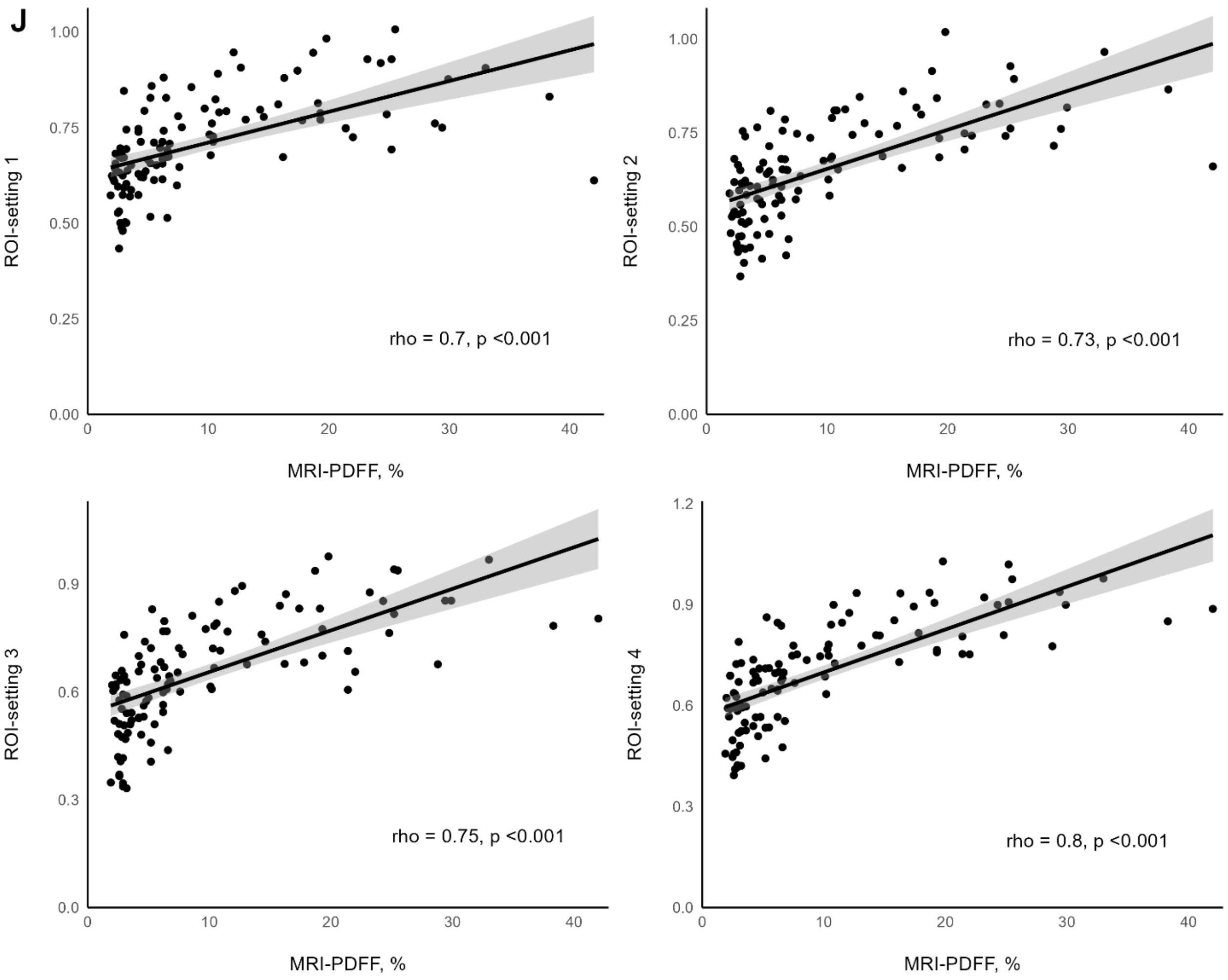
J
ROI-setting 1
rho = 0.7, p <0.001
MRI-PDFF, %
ROI-setting 2
rho = 0.73, p <0.001
MRI-PDFF, %
ROI-setting 3
rho = 0.75, p <0.001
MRI-PDFF, %
ROI-setting 4
rho = 0.8, p <0.001
MRI-PDFF, %

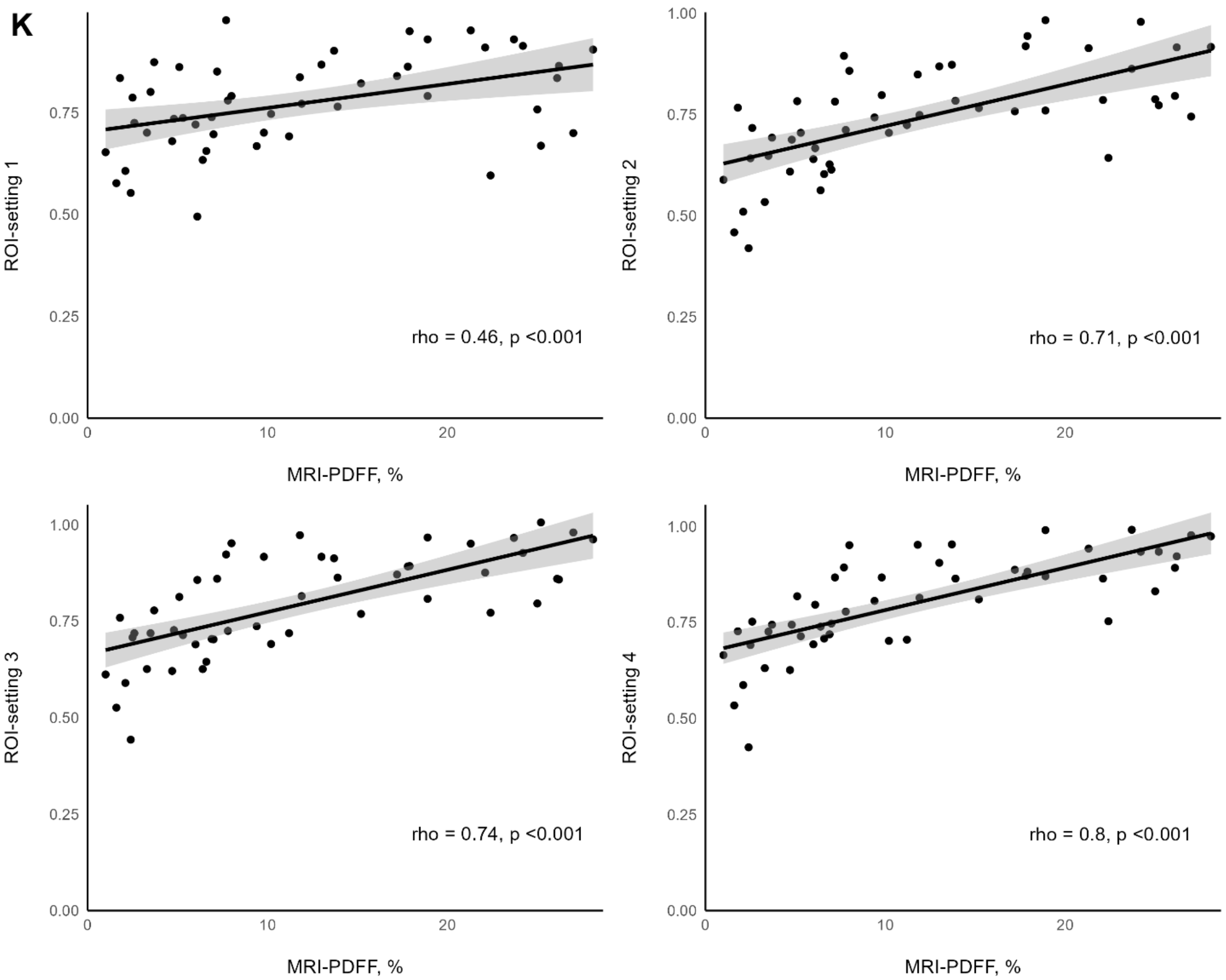

ROI: region of interest; SCD: skin-to-liver capsule distance.
[a]BMI ≥30 for North American participants and BMI ≥28 for Japanese participants.
